# Impairment of myocardial functions and arterial stiffness in patients with lichen planus^☆^^☆☆^

**DOI:** 10.1016/j.abd.2019.07.005

**Published:** 2020-02-12

**Authors:** Leyla Baykal Selcuk, Mursel Sahin, Deniz Aksu Arıca, Asım Orem, Zeynep Karaca Ural, Savaş Yaylı

**Affiliations:** a Department of Dermatology, Karadeniz Technical University, School of Medicine, Trabzon, Turkey; b Department of Biochemistry, Karadeniz Technical University, School of Medicine, Trabzon, Turkey

**Keywords:** Lichen planus, Oral lichen planus, Vascular stiffness

## Abstract

**Background:**

Lichen planus is a chronic inflammatory mucocutaneous disease. Recent studies have suggested that it is associated with an increased risk of cardiovascular comorbidities.

**Objective:**

The purpose of this study was to assess and compare arterial stiffness and cardiovascular hemodynamics in patients with lichen planus and a healthy control group.

**Methods:**

Fifty-five patients with lichen planus and 42 healthy controls were enrolled. All patients underwent echocardiographic examination, and arterial stiffness was measured using applanation tonometry.

**Results:**

No statistically significant difference was determined between the patient and control groups in terms of arterial stiffness, but stiffness was markedly higher in patients with erosive lichen planus compared to the control group and other patients (*p* = 0.006, and *p* = 0.023, respectively). Moderate positive correlation was determined between duration of disease and arterial stiffness. Impairment of systolic and diastolic functions was also determined in patients with lichen planus compared to the control group (*p* < 0.001, and *p* = 0.005, respectively).

**Study limitations:**

Relatively low number of patients.

**Conclusion:**

The positive correlation observed between duration of disease and arterial stiffness in patients with lichen planus suggests that these patients should be followed-up in terms of cardiovascular risk in the presence of resistant and long-term disease, particularly in case of erosive lichen planus.

## Introduction

Lichen planus (LP) is a chronic inflammatory mucocutaneous disease with a prevalence of 0.6% to 1.2% of the population.^1^ The disease is thought to be caused by cell-mediated immunological responses and reactive T lymphocytes are the effector cells causing destruction of keratinocytes.^2^ The duration of the disease varies with the type of LP. The form accompanied by only cutaneous involvement exhibits rapid onset and can improve within one year, while those involving the oral mucosa and nails tend to be more persistent. In particular, erosive and ulcerative oral LP can be lifelong.^3^ Recent studies suggest that LP is associated with increased prevalences of cardiovascular diseases (CVD) due to chronic systemic inflammation.^2^, ^4^, ^5^ Studies have also shown that patients with LP have higher prevalence of metabolic syndrome, dyslipidemia, hyperglycemia, and hypertension. In addition, chronic inflammation leads to endothelial dysfunction, the early stage of atherogenesis.^2^, ^4^, ^6^

Arterial stiffness consists of chronic inflammation and damage to the endothelial function, to structural elements in the arterial wall including muscle, and is a known risk factor for CVD.^7^ Hypertension, hyperlipidemia, older age, diabetes mellitus, chronic heart failure, central obesity, smoking, kidney disease, Coronary Artery Disease (CAD), and stroke are all associated with stiffness.^8^ Of the different methods used to measure arterial stiffness, carotid-femoral pulse wave velocity (PWV) and the aortic Augmentation Index (AIx) are non-invasive and also the gold standard techniques for measurement.^8^, ^9^

Hyperhomocysteinemia, insulin resistance, dyslipidemia, obesity, hypertension and increased serum high-sensitivity C-Reactive Protein (hs-CRP), are risk factors for atherosclerosis and vascular diseases.^2^ Information concerning the relation between LP and arterial stiffness is limited, and stiffness was measured with echocardiographic examination in only one study.

The aim of this study was to evaluate arterial stiffness using the gold standard method, cardiovascular hemodynamics, and associated inflammatory markers in LP patients.

## Methods

### Study design

**Figure 1:**
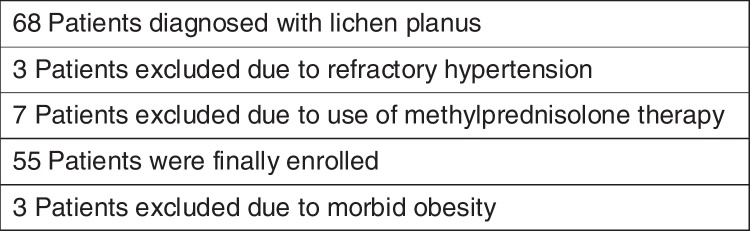
Numbers of patients excluded during establishment of the study population.

This study was performed to evaluate arterial stiffness, associated inflammatory markers and cardiac hemodynamics in patients with LP. The patients and controls were matched in terms of age and sex. We enrolled all patients with LP who admitted to our Department of Dermatology over 18 months. Inclusion criterias were age over 18 years and clinical and histopathological diagnosis of LP. Patients with refractory hypertension, peripheral arterial disease, CAD, renal failure, or morbid obesity, and using such drugs as prednisolone and lipid lowering agents were excluded (Fig. 1). Sixty-eight patients were diagnosed with LP during the study period, and 55 were finally enrolled as the patient group. Nineteen patients had cutaneous disease, 24 mucocutaneous disease and 12 had mucosal lesions only. The control group consisted of 42 individuals. These had no known cardiovascular diseases. All were also free of any inflammatory disease. The source population was the same for the patients and controls.

### Collection of data

Patients and controls signed informed consent forms. Demographic, clinical and laboratory data registered onto study forms by a dermatologist. Blood pressure was measured after seated for at least 5 min. Arterial stiffness and echocardiographic examination were assessed by a single experienced cardiologist to investigate the cardiovascular profile in each case. Blood samples were collected after an overnight fast of 12 h. Levels of total cholesterol, triglyceride and glucose were determined using enzymatic methods. Erythrocyte Sedimentation Rate (ESR) values were determined using the classic Westergren method. Homocysteine levels were assessed using immunonephelometry. High density lipoprotein cholesterol (HDL-C) and low-density lipoprotein cholesterol (LDL-C) were measured with selective detergent methods. hs-CRP levels were assessed with immunonephelometry (Dade Behring, BN II, Marburg, Germany).

### Echocardiographic examination

Echocardiography was performed at rest with an echocardiographic device (Vivid 7, GE Medical Systems, Milwaukee, Wis., USA). Left atrium (LA) and left ventricle (LV) dimension, LV ejection fraction (EF), mitral inflow pattern, and tissue Doppler imaging findings were obtained in line with the current recommendations of the American Society of Echocardiography.^10^ EF was evaluated with the modified biplane method (modified Simpson's rule). The pulsed Doppler sampling volume was evaluated between the tips of the mitral valve leaflets to get maximum filling velocities. Early (E) and late (A) transmitral flow velocities were expressed in meters per second and deceleration time (DT) in milliseconds, and the ratio of early-to-late peak velocities (E/A) was also determined. Peak systolic (S’) and early and late diastolic (e’ and a’) myocardial segment velocities were measured with tissue Doppler imaging of the LV free wall from the apical 4-chamber view. Values for 3 separate cardiac cycles were averaged for all parameters. E/A and E/e’ were calculated for evaluating LV diastolic dysfunction (DD). The myocardial performance index (MPI/Tei Index) including both systolic and diastolic time intervals was also calculated in order to assess global cardiac dysfunction.^11^

### Arterial stiffness measurement

Arterial wall properties, wave reflection PWV, augmentation pressure (AP), and Alx were assessed using applanation tonometry (SphygmoCor, AtCor Medical, Sydney, Australia).^12^ Consecutive registrations of pulse waves of carotid and femoral artery, and the time shift of the appearance of waves at the first and the second sites were calculated. The distance between carotis and femoral pulses was measured on the body surface in order to determine aortic PWV in meters per second. AP was defined as maximum systolic blood pressure minus pressure at the inflection point. AIx was defined as AP divided by pulse pressure. Higher AIx values indicate increased wave reflection from the periphery or earlier return of the reflected wave as a result of increased PWV. AIx is dependent upon the elastic properties of the arteries. Since AIx is influenced by heart rate, we used an index normalized for a heart rate of 75 bpm (AIx@75).^13^ All measurements were performed by the same individual. Mean measurements for 10 cardiac cycles were calculated. Measurements were repeated in case of extreme values.

### Ethical approval

This study was approved by the local ethical committee of Karadeniz Technical University (2016/173) with protocol number of 5554.

### Statistical analyses

Data analysis was performed on SPSS 23.0 software. Results were expressed as number (*n*) and percentage (%) for descriptive data. Chi square analysis was used for comparison for categorical variables. Normal distribution was assessed using the Kolmogorov-Smirnov test. Student's *t* test was used to compare normally distributed constant variables, and Mann–Whitney *U* test to compare non-normally distributed variables. Correlation analysis was performed using Spearman's correlation test. We used linear regression analyze to compare the impact of age, disease duration, CRP levels, and the erosive form of LP on arterial stiffness. A *p*-value <0.05 was considered statistically significant.

## Results

Fifty-five patients (31 women, 24 men, mean age 47.84 ± 13.55 years) and 42 control subjects (21 women, 21 men, and mean age 45.57 ± 8.91 years) were included in the study. Involvement was in the trunk in 28 patients (51%), in the extremities in 40 (73%), in the oral mucosa in 37 (67%), in the genital mucosa in 8 (15%), and in the nails in 3 (6%). White reticular lesions were present in 25 (68%) of the patients with oral mucosa involvement and erosions were observed in 12 (32%). The mean disease duration was 4.09 ± 4.46 years. Mean duration of disease was significantly higher in patients with oral erosive LP (8.16 ± 4.19 years) than in the other patients (2.96 ± 3.86 years) (*p* < 0.001). Clinical and laboratory data for the study population are shown in table 1. Body mass index was significantly higher in the patients than in the controls (*p* = 0.02). No statistically significant differences in other characteristics and cardiovascular comorbidities were observed between the patient and control groups. Inflammatory markers such as CRP and ESR were significantly higher in patients with LP (*p* = 0.049 and *p* = 0.044, respectively), while no difference was determined in other laboratory parameters.

**Table 1:** Clinical characteristics, cardiovascular comorbidities and laboratory data of patients with lichen planus and the controls

	Patients (*n* = 55)	Control (*n* = 42)	*p*-Value
*Age (years)*	47.84 ± 13.5	45.57 ± 8.9	0.325
*Gender (n)*			0.676
Male	24	21	
Female	31	21	
*Body mass index (kg/m*^*2*^*)*	29.76 ± 4.97	27.65 ± 3.87	0.026
*Hypertension, n*	3	3	1.000
*Diabetes mellitus, n*	2	0	0.504
*Alcohol, n*	1	1	1.000
*Fasting plasma glucose, mg/dL*	95.15 ± 19.85	96.31 ± 12.21	0.069
*Total cholesterol, mg/dL*	212.54 ± 63.29	201.53 ± 54.88	0.469
*Triglycerides, mg/dL*	148.57 ± 106.82	145.31 ± 68.45	0.746
*HDL-cholesterol, mg/dL*	54.26 ± 14.47	49.31 ± 10.62	0.123
*LDL-cholesterol, mg/dL*	135.93 ± 47.07	133.14 ± 37.81	0.740
*CRP, mg/dL*	0.40 ± 0.54	0.28 ± 0.44	0.049
*ESR, (mm/s)*	14.28 ± 10.97	9.12 ± 5.76	0.044
*Homocysteine, mg/dL*	12.22 ± 3.92	12.59 ± 7.88	0.469
*Hemoglobin, g/L*	13.95 ± 1.30	13.85 ± 1.16	0.717
*Leukocyte, ul*	7532.31 ± 1681.45	7133.33 ± 1399.04	0.221
*Platelet, uL*	258,333.33 ± 52,642.98	244,421.43 ± 78,063.71	0.176
*Systolic BP, mm/hg*	120.65 ± 16.37	121.19 ± 13.29	0.847
*Diastolic BP, mm/hg*	77.41 ± 10.76	77.86 ± 8.47	0.652

Transthoracic echocardiographic measurements and arterial stiffness findings in the study population are shown in table 2. MPI and E velocity and the E/A ratio, markers of diastolic dysfunction, differed significantly between the LP patients and the controls (*p* < 0.001, *p* < 0.001 and *p* = 0.005, respectively). No statistically significant difference in arterial stiffness measurements was observed between the patient and control groups, although stiffness was significantly higher in patients with erosive oral LP (10.32 ± 1.26) compared to the control group (9.09 ± 1.40) and to the other LP patients (9.12 ± 1.75) (*p* = 0.009 and *p* = 0.023, respectively) (Table 3). Mean ESR levels were 21.50 ± 13.40 mm/s in the erosive LP patients and 12.21 ± 9.37 mm/s in the other patients, and CRP levels were significantly higher in the erosive LP group (0.78 ± 0.89 mg/dL) compared to the other LP patients (0.29 ± 0.33 mg/dL) (*p* = 0.01 and *p* = 0.002, respectively).

**Table 2:** Echocardiographic and arterial stiffness parameters in the patients and controls

	Patients (*n* = 55)	Controls (*n* = 42)	*p*-Value
LVSD (mm)	29.85 ± 3.58	29.76 ± 2.73	0.889
LVDD (mm)	46.09 ± 2.70	46.16 ± 3.12	0.371
EF (%)	62.75 ± 2.81	62.81 ± 2.82	0.918
IVS (mm)	10.40 ± 0.97	10.09 ± 1.10	0.169
PW (mm)	9.89 ± 0.96	9.61 ± 0.99	0.260
LA (mm)	33.98 ± 2.86	34.21 ± 2.99	0.769
E (m/s)	0.67 ± 0.16	0.82 ± 0.14	<0.001
A (m/s)	0.66 ± 0.21	0.65 ± 0.17	0.754
DT	208.24 ± 46.00	193.52 ± 34.67	0.231
E/A	1.11 ± 0.42	1.33 ± 0.34	0.005
S’	0.92 ± 0.23	0.83 ± 0.21	0.053
e’ (cm/s)	1.16 ± 0.36	1.29 ± 0.40	0.219
a’(cm/s)	1.06 ± 0.31	1.15 ± 0.37	0.284
E/e’	6.4 ± 0.33	6.8 ± 0.23	0.116
MPI	0.51 ± 0.09	0.43 ± 0.05	<0.001
Aortic-PWV (m/s)	9.46 ± 1.7	9.09 ± 1.40	0.256
AIx (%)	24.12 ± 11.39	22.26 ± 11.31	0.425
AIx@75 (%)	22.90 ± 10.71	20.78 ± 11.05	0.366
AP	8.54 ± 5.45	7.95 ± 5.36	0.574

LVSD, left ventricular end systolic diameter; LVDD, left ventricular end diastolic diameter; IVS, interventricular diameter.

**Table 3:** Echocardiographic and arterial stiffness parameters in erosive LP patients and the controls

	Erosive LP (*n* = 55)	Control (*n* = 42)	*p*-Value
LA (mm)	35.25 ± 2.30	34.21 ± 2.99	0.274
E (m/s)	0.65 ± 0.11	0.82 ± 0.14	<0.001
A (m/s)	0.71 ± 0.21	0.65 ± 0.17	0186
DT	189.83 ± 37.19	193.52 ± 34.67	0.750
E/A	0.99 ± 0.30	1.33 ± 0.34	0.004
S’	0.95 ± 0.22	0.83 ± 0.21	0.102
e’ (cm/s)	1.05 ± 0.23	1.29 ± 0.40	0.086
a’	1.28 ± 0.31	1.15 ± 0.37	0.225
E/e’	0.66 ± 0.22	0.68 ± 0.23	0.687
MPI	0.51 ± 0.57	0.43 ± 0.05	0.001
Aortic-PWV (m/s)	10.32 ± 1.26	9.09 ± 1.40	0.009
AIx (%)	29.00 ± 7.21	22.26 ± 11.31	0.051
AIx@75 (%)	27.08 ± 8.46	20.78 ± 11.05	0.094
AP (mmHg)	10.41 ± 3.94	7.95 ± 5.36	0.146

Correlation was analyzed between duration of disease and arterial stiffness, echocardiographic and laboratory parameters (Table 4). Positive correlation was determined between duration of disease and arterial stiffness markers. The dependent effects of erosive LP, duration of disease, patient age and CRP levels on arterial stiffness were assessed using regression analysis and increased duration of disease and CRP levels were observed to constitute risks for development of stiffness (Table 5).

**Table 4:** Correlations between duration of disease and arterial stiffness, echocardiographic and laboratory parameters

	*r*	*p*-Value
PWV	0.447	0.001
AP	0.352	0.013
AIX	0.331	0.008
AIX 75	0.319	0.017
ESR	0.286	0.036
Leukocyte	−0.306	0.024
Age	0.168	0.221
BMI	−0.173	0.206
Glucose	0.091	0.509
CRP	0.003	0.985
Systolic BP	0.151	0.277
Diastolic BP	0.143	0.303

**Table 5:** Findings of linear regression analysis performed to determine the independent predictors of PVW in patients with LP

	*β*	*p*-Value
Disease duration (years)	2.133	0.003
Age	0.028	0.071
Erosive LP	0.916	0.195
CRP (mg/dL)	1.501	0.014

## Discussion

Arterial stiffness in our study was significantly higher in the erosive type of LP, which progresses with resistant erosions in the mouth and/or genital mucosa. Positive correlation was also determined between duration of disease and arterial stiffness. CRP and ESR levels were significantly higher in patients with LP than in the control group, and were also significantly higher in patients with erosive LP than in patients without erosive LP. Based on these findings, we conclude that arterial stiffness levels increase in the erosive oral form of LP, and that a prolonged duration of disease increases arterial stiffness.

LP is a disease that progresses with chronic inflammation, similarly to psoriasis vulgaris, and in which T cell-mediated immune response accompanied by endothelial dysfunction is present. While T helper lymphocytes predominate in dermal infiltrate in early lesions, CD8 + cytotoxic T cell infiltration is observed in late lesions. Activated T cells cause induction of cytokines and inflammatory cells such as Tumor Necrosis Factor-α (TNF-α), Interleukin-2 (IL-2), and Interferon-γ (IFN-γ), leading to keratinocyte damage.^3^ Additionally, elevated cytokines such as Interleukin (IL)-2, IL-6 and TNF-α in patients produce metalloproteinases that reduce the content of the aortic intima, and may thus promote increased arterial stiffness.^14^ Increased reactive oxygen species and lipid peroxides have also been implicated in the pathogenesis of LP.^15^ Increases of 50% in rates of CVD such as myocardial infarction and stroke have been shown in individuals with various chronic inflammatory diseases, such as psoriasis, rheumatoid arthritis, and inflammatory bowel disease, compared to the general population.^16^ Similarly, various studies have examined the relation between LP and cardiovascular and metabolic comorbidities, and increased prevalences of metabolic syndrome, dyslipidemia and hypertension have been reported.^2^, ^4^, ^6^ In a previous study of ours, we determined an increased prevalence of metabolic syndrome in LP, and particularly in the oral form.^17^

Diastolic dysfunction can be the first sign of cardiovascular and metabolic diseases isovolumic relaxation time (IVRT), early to late ventricular filling velocities ratio, and early flow deceleration time were used to asses diastolic function on echocardiography.^18^ Left ventricular diastolic dysfunction is a clinically significant condition. Diastolic dysfunction can by itself give rise to symptoms and findings of heart failure even if systolic functions are normal. The diastolic functions of the heart depend on complex, inter-related factors. The principal causes of diastolic dysfunction are advanced age, hypertension and ischemic heart disease; although several diseases progressing with systemic inflammation can also affect left ventricular diastolic functions. Previous studies have determined impairment of cardiac functions in diseases involving diffuse inflammation, such as RA and psoriasis.^19^, ^20^ Similarly, one study of left ventricular systolic and diastolic functions in LP patients determined significant differences in patients compared to a control group.^14^ In our study, diastolic dysfunction was similarly higher in the patient group. Although the E/A ratio were lower than in the control group, it was still within normal ranges in both groups. This indicates that the existing impairment is at a very low level. Values indicating diastolic dysfunction, such as DT, LA dimension and E/e, being similar to those in the control group supports this idea. In contrast to Koseoglu et al., when erosive patients, with greater inflammation and duration of disease, were analyzed separately in our study, similar findings were also obtained. Considering these findings together, LP appears to have a mild effect on left ventricular diastolic dysfunction. MPI was evaluated in our study since it reflects the global function of the heart at the subclinical level. In agreement with previous studies, MPI was significantly higher in the LP patients.^14^ A similar effect was determined in the erosive patient group.

Evaluation of arterial stiffness and the elastic features of the aorta through wave reflections is becoming widely used in the clinical assessment of patients with inflammatory diseases.^21^ Arterial stiffness is a systemic condition with a close impact on cardiac functions, and larger wave reflections also contribute to the risk of CVD.^16^, ^21^, ^22^, ^23^ Carotid–femoral PWV is an indicator of elastic-type aortic stiffness, while AIx is a marker of wave reflection. Carotid-femoral PWV and AIx are the gold standard methods. Increased arterial stiffness adversely affects systolic and diastolic functions of the myocardium and cardiac structure.^24^

Chronic systemic inflammation can lead to atherosclerosis, which progresses as long as the inflammation persists. The existing inflammation and endothelial dysfunction in LP are thought to be capable of increasing arterial stiffness, resulting in arterial wall damage.^14^, ^25^ A previous study showed increased arterial stiffness and myocardial index values in patients with LP compared to a control group. In that study, Koseoglu et al. evaluated arterial stiffness using echocardiography. Significantly high aortic stiffness was determined independently of type of disease compared to the control group.^14^ In contrast to their study, we observed no significant difference in arterial stiffness between our patient and control groups, but significantly greater stiffness was determined in patients with erosive oral LP. In agreement with other studies, we determined positive correlation between duration of disease and arterial stiffness, evaluated using PWV. PWV and Aix evaluation of stiffness is a more sensitive and gold standard method than echocardiography. In contrast to other studies, all arterial stiffness parameters were similar in our patient and control groups, but significantly increased arterial stiffness was observed when erosive patients were assessed. On the basis of these findings, we concluded that arterial stiffness in LP is seen more in patients with a greater duration of disease and greater inflammation. The marked elevation in arterial stiffness despite the absence of any pronounced impairment of cardiac functions in the patient group suggests that inflammation is more effective at the vascular cell level than at the myocardial level. Further studies at the cellular level are needed to for more detailed clarification of this.

Homocysteine is known to exhibit proatherogenic effects and may play a role in endothelial damage.^18^ CRP is deposited in the arterial intima in early atherosclerotic lesions, induces an inflammatory and atherogenic phenotype in endothelial cells, and stimulates vascular smooth muscle cell migration and proliferation.^26^ Saleh et al. reported significantly higher homocysteine, fibrinogen and hs-CRP in LP patients than in controls.^2^ In our study, however, homocysteine levels were similar, but CRP and ESR values were higher in the patient group, and especially in patients with erosive LP.

The relatively low numbers of patients in our study should be regarded as a limitation. Further studies with greater numbers are now needed to support our conclusions.

## Conclusion

We determined significant correlation between arterial stiffness and duration of disease in LP patients, and particularly those with erosive LP, a resistant form. Based on our findings, the risk of cardiovascular disease is greater in subjects with a longer duration of disease and with a resistant form, and these patients must be placed under closer monitoring.

## Financial support

This study was supported by the Scientific Research Unit of Karadeniz Technical University, under project number 5554.

## Authors’ contributions

Leyla Baykal Selcuk: Statistic analysis; approval of the final version of the manuscript; conception and planning of the study; elaboration and writing of the manuscript; obtaining, analysis, and interpretation of the data; effective participation in research orientation; intellectual participation in the propaedeutic and/or therapeutic conduct of the studied cases; critical review of the literature; critical review of the manuscript.

Mursel Sahin: Conception and planning of the study; obtaining, analysis, and interpretation of the data; effective participation in research orientation; critical review of the manuscript.

Deniz Aksu Arıca: Conception and planning of the study.

Asım Orem: Conception and planning of the study.

Zeynep Karaca Ural: Statistic analysis; obtaining, analysis, and interpretation of the data; effective participation in research orientation.

Savaş Yaylı: Conception and planning of the study; critical review of the manuscript.

## Conflicts of interest

None declared.
